# Robust Detection of Cracked Eggs Using a Multi-Domain Training Method for Practical Egg Production

**DOI:** 10.3390/foods13152313

**Published:** 2024-07-23

**Authors:** Yuxuan Cheng, Yidan Huang, Jingjing Zhang, Xuehong Zhang, Qiaohua Wang, Wei Fan

**Affiliations:** 1 College of Engineering, Huazhong Agriculture University, Wuhan 430070, China; hxwxss@webmail.hzau.edu.cn (Y.C.); zhanguu0910@163.com (J.Z.); zxh666666@webmail.hzau.edu.cn (X.Z.); wqh@mail.hzau.edu.cn (Q.W.); 2 College of Informatics, Huazhong Agriculture University, Wuhan 430070, China; huangyidan@webmail.hzau.edu.cn

**Keywords:** cracked egg, unknown egg test domain, multi-domain training, MMD, robust, efficient

## Abstract

The presence of cracks reduces egg quality and safety, and can easily cause food safety hazards to consumers. Machine vision-based methods for cracked egg detection have achieved significant success on in-domain egg data. However, the performance of deep learning models usually decreases under practical industrial scenarios, such as the different egg varieties, origins, and environmental changes. Existing researches that rely on improving network structures or increasing training data volumes cannot effectively solve the problem of model performance decline on unknown egg testing data in practical egg production. To address these challenges, a novel and robust detection method is proposed to extract max domain-invariant features to enhance the model performance on unknown test egg data. Firstly, multi-domain egg data are built on different egg origins and acquisition devices. Then, a multi-domain trained strategy is established by using Maximum Mean Discrepancy with Normalized Squared Feature Estimation (NSFE-MMD) to obtain the optimal matching egg training domain. With the NSFE-MMD method, the original deep learning model can be applied without network structure improvements, which reduces the extremely complex tuning process and hyperparameter adjustments. Finally, robust cracked egg detection experiments are carried out on several unknown testing egg domains. The YOLOV5 (You Only Look Once v5) model trained by the proposed multi-domain training method with NSFE-MMD has a detection mAP of 86.6% on the unknown test Domain 4, and the YOLOV8 (You Only Look Once v8) model has a detection mAP of 88.8% on Domain 4, which is an increase of 8% and 4.4% compared to the best performance of models trained on a single domain, and an increase of 4.7% and 3.7% compared to models trained on all domains. In addition, the YOLOV5 model trained by the proposed multi-domain training method has a detection mAP of 87.9% on egg data of the unknown testing Domain 5. The experimental results demonstrate the robustness and effectiveness of the proposed multi-domain training method, which can be more suitable for the large quantity and variety of egg detection production.

## 1. Introduction

Eggs, as a high-protein and nutritionally rich food, are one of the most commonly consumed dietary items among the general population [1]. Due to the physical properties of eggshells and their internal structural features [2], they are susceptible to damage during various processes such as farming, production, and transportation [3]. The presence of damaged eggs can adversely affect egg quality, leading to a reduced shelf life. This not only increases the risk of bacterial contamination in human consumption [4] but also diminishes the operational efficiency of businesses.

In general, cracked egg detection relies on percussion vibration [5,6,7] and machine vision detection [8]. Percussion vibration primarily utilizes sound signals and vibration sensor data to analyze whether the tested samples exhibit surface cracks. Botta et al. [9], utilizing the theory of resonant inspection (RI), extracted the acoustic features of eggs and achieved the detection of cracked eggs by combining support vector machines (SVM). However, percussion vibration systems are affected by ambient sounds, so their accuracy decreases in actual industrial environments.

In recent years, with the rapid advancement of computer vision, notably represented by Convolution Neural Network (CNN) and Visual Transformers (ViT) [10,11,12], machine vision-based methods for cracked egg detection have made significant breakthroughs. Many research papers often utilize image data collected in laboratory settings or single industrial environments for model training, achieving remarkable results on the in-domain egg data [13,14]. Xiong et al. [15] utilized various feature parameters and Support Vector Machines (SVM) for classifying cracked and non-cracked eggs. They also employed brightness reduction and region labeling techniques to preprocess egg surface images. Experimental results showed that the model achieved an accuracy of 97.5%. Bao et al. [8] employed a negative LOG (Laplace–Gaussian) operator and hysteresis threshold algorithm to enhance crack features under backlighting conditions. Experimental results indicated a recognition rate of 92.5%. Huang et al. [16] improved the YOLOv5 model by incorporating CA, integrating BiFPN and GSConv with Neck, to enable the detection and tracking of damaged, unwashed eggs on the production line.

**Figure 1:**
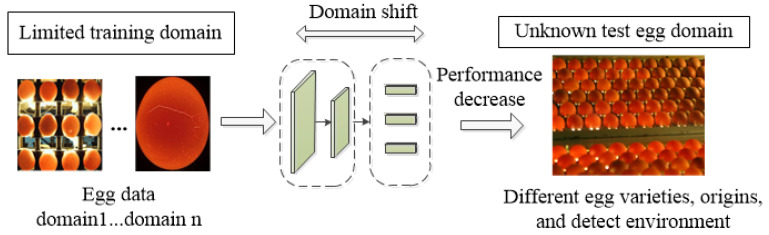
Domain shifting variations in egg detection performance.

Traditional cracked egg detection methods based on deep learning have achieved awesome progress in single in-domain egg data. However, the deployment environment for visual systems and the varieties and origins of egg samples are often changed in practical egg production. As shown in Figure 1, the performance of trained detection models often deteriorates on unknown egg detection scenarios. Many studies have shown that supervised data-driven deep learning models experience significant accuracy degradation when confronted with domain shifts between the training and real-world deployment scenarios [17,18,19]. For instance, Mohanty et al. [20] achieved a 99.34% accuracy rate in classifying crop diseases using the GoogLeNet model but faced significant challenges when handling out-of-distribution data, resulting in accuracies of 31.4% and 31.69% in two target domain datasets. In the field of egg detection, several cracked egg datasets can be acquired during the research phase, yet the actual production process faces a large quantity of unknown egg data. The performance of deep learning models trained to assume that the training egg data and target egg data share similar features will inevitably decrease.

To address this problem, researchers attempt to improve the generalization of the deep learning model from two aspects. Many works focus on improving the network architectures [21,22], such as utilizing attention mechanisms, regularization, or building a deeper model. However, the efforts are almost meaningless on unknown test egg data in practical production. The complicated network structure design and parameter tuning usually cause the model to overfit the training data. On the other hand, the researchers start with training data. More diverse annotated data can help facilitate the model’s extraction of domain-invariant representations. However, the cost of time and samples prevents the model training data from increasing indefinitely. Some researchers also try to reduce the sample acquisition cost by generating more training samples through the generative model [23,24,25], but it still cannot reduce the multi-object labeling cost in the detection scene of the production line. Nevertheless, blindly adding multi-domain datasets is costly and may lead to feature confusion. It is necessary to judiciously utilize limited multi-domain egg data for training to enhance the model’s generalization ability in unknown cracked egg detection.

Representation learning-based methods are widely used algorithms for addressing domain shifts. Representation learning-based methods aim to facilitate domain-invariant feature representations for samples. MMD (Maximum Mean Discrepancy) [26] is a method for measuring the difference between two related probability distributions and maintaining greater distribution differences, while preserving semantic consistency helps promote the extraction of domain-invariant features [19,27,28]. Inspired by this, a multi-domain training method with NSFE-MMD is proposed. This approach effectively facilitates the extraction of domain-invariant features in limited egg data, thereby enhancing the performance of the defective egg detector on egg data from unknown testing domains.

In this study, egg datasets are captured from two prevalent sample acquisition scenarios (multi-channel production lines and static light box) and diverse egg samples from different origins. The multi-domain training approach with NSFE-MMD is proposed to realize robust detection of unknown egg data coming from practical production. This method is integrated into the original YOLOv5 and YOLOv8 models, which do not need to be tuned or parameter adjusted. Subsequently, the performance of the model is evaluated by cracked egg detection on distinct egg data from different origins and acquisition environments. The manuscript is organized as follows: Section 2 introduces the establishment of the data collection system, sample acquisition, and the proposed methodology. Section 3 outlines the experiments conducted on diverse egg data from unknown testing domains and the discussion of the results. Section 4 discusses the research findings and conclusion.

## 2. Materials and Method

### 2.1. Machine Vision System

In practical production, egg data usually come from different places of origin and acquisition environments. To ensure the dataset’s relevance to the industrial production scene, a significant quantity of eggs was acquired from Wuhan, Qingdao and Nanyang, China. The image acquisition process was carried out in the production line and the static acquisition box at the College of Engineering, Huazhong Agricultural University.

**Figure 2:**
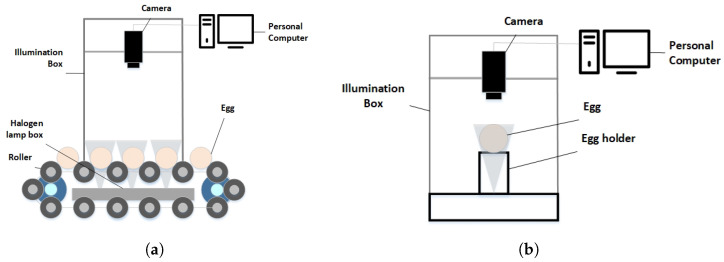
Image acquisition device. (**a**) Dynamic machine vision system. (**b**) Static machine vision system.

The dynamic production line, illustrated in Figure 2a, comprises a three-channel conveyor belt, camera, and light source. This setup has widely demonstrated significant advantages in capturing images depicting various surfaces of eggs [29]. Notably, it enables the transportation of up to 15,000 eggs per hour. Each channel is equipped with numerous rollers driven by motors. As a result, images captured by the production line typically feature 9–12 eggs in each frame. As Figure 2b shows, the second device is a static acquisition box typically used for collecting a small number of static samples. It is equipped with an industrial camera connected to a computer, providing static sample collection scenarios. The static acquisition device usually only captures a single egg in each image.

### 2.2. Multiple Domain Egg Samples Preparation

**Figure 3:**
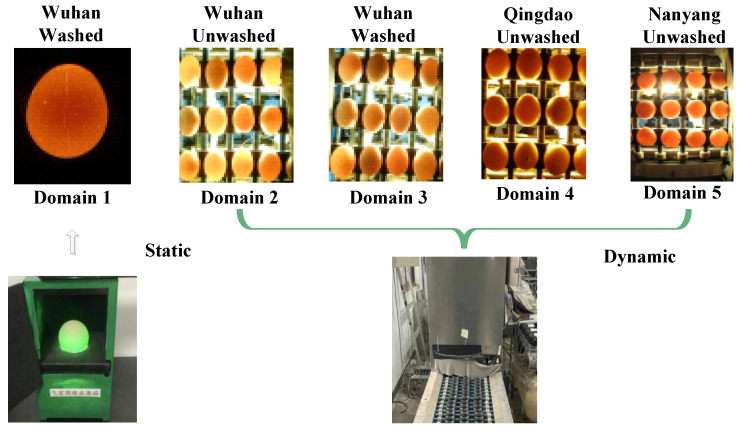
Samples from different domains.

As shown in Table 1, a total of 800 eggs were used in this study from three provinces in China: Wuhan of Hubei, Qingdao of Shandong, and Nanyang of Henan. Shendan, Zhengda, and Wenshi are the brands of eggs. The eggs were acquired from different origins, devices, and environments, and the five egg data have distinct domain shifts to each other, which can be known in Figure 3. During the data collection process on the production line, hours of video are captured using cameras, and a substantial number of training samples through frame extraction at fixed intervals.

Moreover, substantial differences can be observed among egg samples obtained from different origins, environments, and devices. Domain 1, Domain 2, and Domain 3 serve as the known training data for the model. Domain 1 consists of washed eggs from Wuhan, collected in a static acquisition box in a laboratory environment. Domain 2 comprises unwashed eggs from Wuhan, collected through a dynamic production line. Domain 3 comprises washed eggs from Wuhan, also collected through a dynamic production line.

In contrast to Domain 1, the dynamic data capture process in Domain 2 and Domain 3 results in motion blur, and there is a notable discrepancy in the sample count within a single image. Domain 2, which features eggs that have not been cleaned, presents additional interference due to dirt—a factor not present in Domain 1 and Domain 3. Domain 4 and Domain 5 are excluded from the model training phase; they are earmarked as unknown testing domains. Domain 4 and Domain 5 came from different origins with the training data, showing differences in egg varieties and shell transparency.

### 2.3. The Architectures of Multi-Domain Training Method with NSFE-MMD

In practical scenarios demanding a balance between detection efficiency and accuracy, single-stage object detectors are widely favored for cracked egg detection [16,30]. Currently, the cracked egg detection method, built upon an improved object detector, has shown promising results [31]. However, an increase in computational complexity and the number of parameters may impact the recognition performance of the object detector under the egg data with unknown distributions. Excessive parameters are prone to causing model overfitting, and the design of a redundant network structure can also impede detection efficiency. To boost the recognition performance of the model under the egg data of unknown testing domain, a strategy is proposed without requiring alterations to the original model.

**Figure 4:**
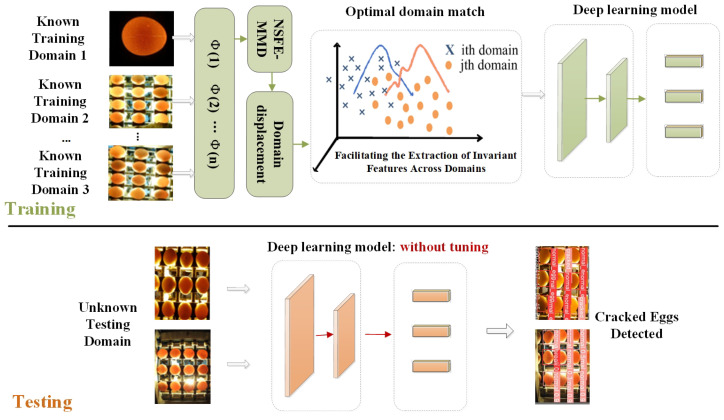
The architectures of robust cracked egg detector with NSFE-MMD.

The diagram of the multi-domain training method with NSFE-MMD is illustrated in Figure 4. Specifically, in the training phase, egg data from different origins are input into the Domain displacement layer to perform optimal domain matching using NSFE-MMD. This is then fed into a general deep learning model (object detector) for training. In the testing phase, facing the egg data of unknown testing domains, the model can achieve good detection results without tuning or parameter adjustment.

### 2.4. Proposed NSFE-MMD

#### 2.4.1. Problem Statement

Compared to the detection methods currently employed in laboratory environments or single scenarios, actual egg production detection often involves dynamic multi-channel processes and variations in factors such as origin and batch. Domain shift between the testing domain egg dataset and the known domain dataset used for training leads to the degradation of model performance. It is assumed that the image domain can be expressed as

(1) D={X,P(X)}

where *X* is the feature space of i.i.d. samples from *D* and P(X) is the marginal probability distribution. The labeled known egg data distribution and the unknown target egg data distribution can be defined as

(2) $$S={\left\{\left(x_{i};\left(y_{i},B_{i}\right)\right)\right\}}_{i=1}^{n}∼{\left(D_{S}^{X}\right)}^{n}$$

(3) $$T={\left\{x_{i^{\prime }}\right\}}_{i=1}^{n^{\prime }}∼{\left(D_{T}^{X}\right)}^{n^{\prime }}$$

where *S* and *T* represent the data distributions of the egg data of the known training domain and the egg data of the unknown testing domain, respectively. *n* is the number of samples in the known egg data domain, and $n^{\prime }$ is the number of samples in the egg data of the unknown testing domain. $\left(y_{i},B_{i}\right)$ are the label information. $D_{S}$ and $D_{T}$ are the probability distributions of the known egg data domain and target egg data domain, respectively.

#### 2.4.2. Algorithm Principle of NSFE-MMD

The NSFE-MMD method is proposed to utilize the known egg data domain to extract as many domain-invariant features as possible to enhance the model’s performance on the unknown testing egg domain. Computing the domain distance is essential because it identifies the domains with the greatest dissimilarity, which can be exploited as training data to enhance the model’s ability to extract domain-invariant features. By training on the domain pair with the maximum deviation, while maintaining semantic consistency, the model is better equipped to generalize to the dataset with unknown distribution, thus improving its performance on the unknown target egg data domain. The Maximum Mean Discrepancy (MMD) [26] is a method used to measure the difference between two distinct data distributions by mapping the data into a Reproducing Kernel Hilbert Space (RKHS). Here, it is assumed that there are two data distributions, representing the known and target domains, respectively. The sample sets for these distributions are denoted as and *Q*. Using H to represent RKHS, the mapping function, maps X to H. Thus, MMD can be expressed as follows (4):

(4) $$\begin{array}{c} \begin{array}{cc} {\mathrm{MMD}}^{2}\left(X_{s},X_{t}\right)= & \frac{1}{n(n-1)}\sum_{i=1}^{n}\sum_{j=1}^{n}k\left(\phi \left(x_{i}^{s}\right),\phi \left(x_{j}^{s}\right)\right)\\ \; & -\frac{2}{mn}\sum_{i=1}^{n}\sum_{j=1}^{m}k\left(\phi \left(x_{i}^{s}\right),\phi \left(x_{j}^{t}\right)\right)\\ \; & +\frac{1}{m(m-1)}\sum_{i=1}^{m}\sum_{j=1}^{m}k\left(\phi \left(x_{i}^{t}\right),\phi \left(x_{j}^{t}\right)\right) \end{array} \end{array}$$

- *n* is the number of samples in the first domain.
- *m* is the number of samples in the second domain.
- *i* represents the *i*-th sample in the first domain.
- *j* represents the *j*-th sample in the second domain. $k(\dot{)}$ is the kernel function.

Due to the relatively small domain differences inherent in eggs from various origins, common feature spaces [32] often struggle to estimate the domain discrepancies among samples. A simple nonlinear kernel function has been designed to map samples into the feature space, as shown in Equation (5).

(5) $$k\left(x_{i},y_{i}\right)={\left(x_{i}\cdot y_{i}\right)}^{2}$$

To ensure the comparability of the MMD measures across different data or feature spaces, the calculated data are normalized. The Normalized Squared Feature Estimation-MMD (NSFE-MMD) can be represented as

(6) $${\mathrm{NSFE}-\mathrm{MMD}}^{2}\left(P,Q\right)=\frac{MMD^{2}\left(P,Q\right)}{\max_{\phi ∼H}MMD^{2}\left(\phi ,\phi \right)}$$

Here, *P* and *Q* are two probability distributions, H is the considered feature space, ϕ is a test function within that space, $MMD^{2}\left(P,Q\right)$ is the maximum mean discrepancy between the two distributions, and $\max_{\phi ∼H}MMD^{2}\left(\phi ,\phi \right)$ is the maximum value of the MMD squared over all possible test functions in the given feature space. The previously mentioned multi-origin egg datasets have their pairwise domain distances calculated based on NSFE-MMD.

Table 2 shows the domain distance predicted by NSFE-MMD between the collected egg domains in Section 2. The difference between Domain 1 and Domain 3 is significantly higher than that of other combinations. The optimal match domain pair can be obtained by maximum distance and maintain semantic consistency.

## 3. Experiments and Results

### 3.1. Model Metrics

The training results of the YOLOv5 and YOLOv8 models are assessed using mAP (mean average precision), representing the average value of all AP (average precision). AP is the mean of the PR (precision–recall) curve, reflecting the model’s accuracy in identifying cottonseeds. The relevant terms are defined as follows:

(7) $$AP=\sum_{0}^{1}p(r)dr\;$$

(8) $$Precision=\frac{TP}{TP+FP}$$

(9) $$Recall=\frac{TP}{TP+FN}$$

(10) $$mAP=\frac{\sum AP}{N}$$

Precision indicates the model’s accuracy in identifying positive samples;

Recall reflects the model’s ability to correctly classify all positive samples;

TP—true positive samples;

FP—false positive samples;

FN—false negative samples;

*N*—the number of samples.

### 3.2. Implementation Details

The training configuration is outlined in Table 3. A batch size of 16 is adopted for training the network. The optimization of the network model parameters is achieved through Stochastic Gradient Descent (SGD). The starting values for the learning rate, momentum, weight decay, and additional parameters are in line with the standard settings found in YOLOv5 and YOLOv8. Adjustments to the learning rate are conducted via the cosine annealing method. This training is carried out on a GPU, extending over 100 epochs.

In these experiments, to evaluate the effectiveness of the proposed method, the data are selected from Domain 1 (Wuhan, washed, static), Domain 2 (Wuhan, unwashed, dynamic), and Domain 3 (Wuhan, washed, dynamic) for training. To ensure the fairness of the experiment, Domain 4 (Qingdao, unwashed, dynamic) and Domain 5 (Nanyang, unwashed, dynamic) are designated, encompassing samples from different origins and acquisition devices, as the testing domains for the model. Following 100 epochs of training on the training dataset, the model’s final weights (last.pt) are utilized for testing on Domain 4 and Domain 5.

### 3.3. Training Curve Analysis

**Figure 5:**
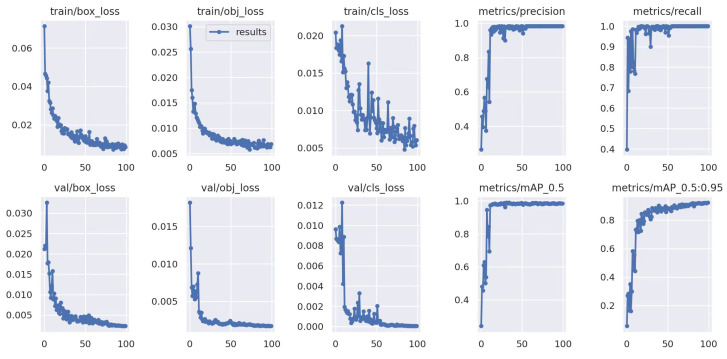
YOLOv5 testing on the known Domain 1 with single-domain training method, which is trained on Domain 1.

The cracked egg detection experiments are conducted on different egg data domains. The iterative curve of YOLOv5 is shown in Figure 5. Similar to usual in-domain detection, the training and test egg data are both Domain 1. The results demonstrate that classical YOLOv5 exhibits strong performance when trained and tested on in-domain egg data.

**Figure 6:**
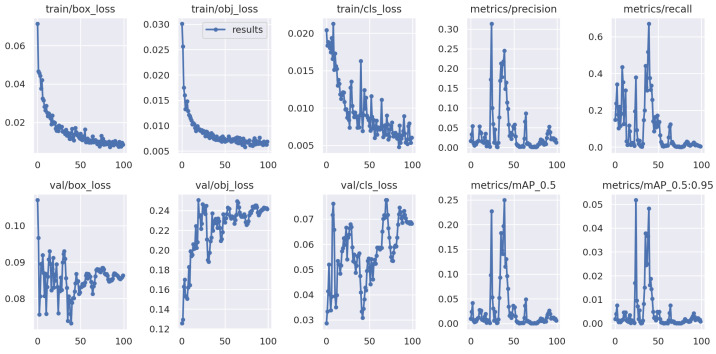
YOLOv5 testing on the unknown Domain 4 with single-domain training method, which adopts Domain 1 as training data.

Figure 6 illustrates the iterative performance curve when testing on unknown Domain 4 (Qingdao, unwashed, dynamic), which uses single Domain 1 (Wuhan, washed, static) as the training data. In contrast to the results shown in Figure 5, unacceptable accuracy losses are observed when testing on unknown test egg data in different origin and detection environments. This observation reinforces our conclusion that the model’s performance significantly deteriorates when presented with practical egg data in different varieties, origins, or environments.

**Figure 7:**
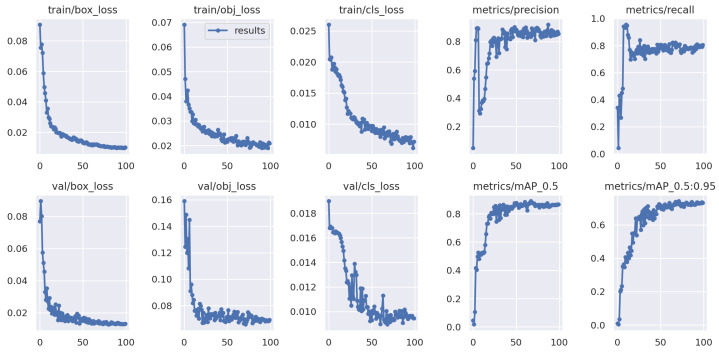
Testing YOLOv5 on the unknown Domain 4, with randomly selected Domain 1 and Domain 2 as training data.

Figure 7 showcases training on Domain 1 (Wuhan, washed, static) and Domain 2 (Wuhan, unwashed, dynamic), where the loss function stabilizes and rapidly converges during training. Similarly, there is an oscillation in performance on the egg data of the unknown testing domain, but it eventually recovers to a good level.

**Figure 8:**
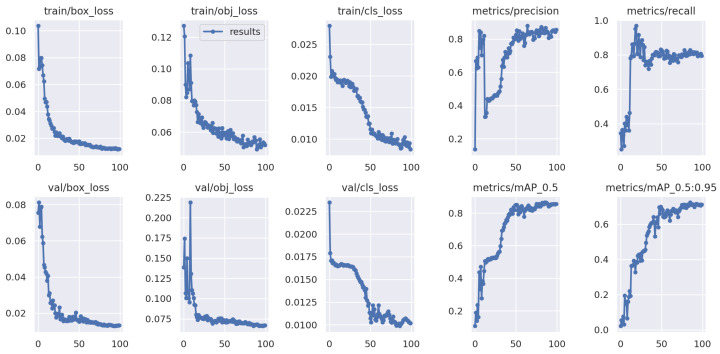
Testing YOLOv5 on the unknown Domain 4, with randomly selected Domain 2 and Domain 3 as training data.

Figure 8 illustrates the curves for training on Domain 2 (Wuhan, unwashed, dynamic) and Domain 3 (Wuhan, washed, dynamic) as the training data. The loss function consistently decreases and converges on both the training set and the unknown testing data of Domain 4 (Qingdao, unwashed, dynamic). However, there is some oscillation in performance on the egg data of the unknown testing domain, which takes a longer duration to recover to satisfactory levels.

**Figure 9:**
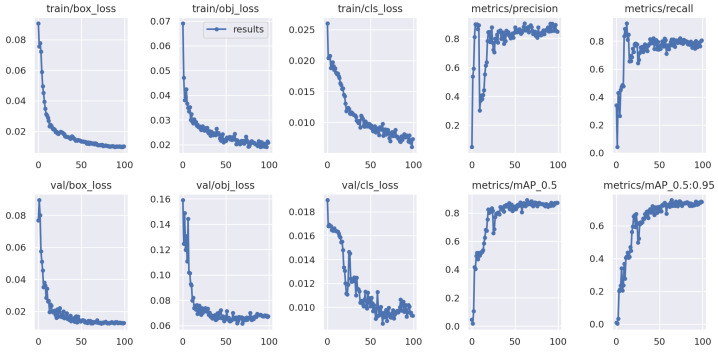
YOLOv5 testing on the unknown Domain 4 with multi-domain training method with NSFE-MMD, which adopts Domain 1 and Domain 3 as the trained data.

Figure 9 displays the curves when using Domain 1 (Wuhan, washed, static) and Domain 3 (Wuhan, washed, dynamic) as the training set. Upon analyzing various multi-domain training curves, it becomes evident that utilizing domains with the maximum domain distance as training data (Domain 1 and Domain 3) results in the model achieving the highest mean Average Precision (mAP) on the egg data of the unknown testing domain (Domain 4). Larger domain distances facilitate quicker overcoming of temporary accuracy drops in the mid-training phase of multi-domain training, leading to faster convergence to higher overall performance (mAP). However, in the mid to late stages of training, the model acquires superior domain-invariant representations from multi-domain data, resulting in enhanced overall performance.

**Figure 10:**
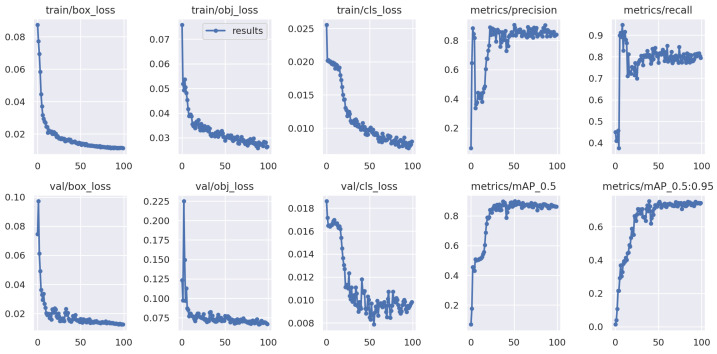
YOLOv5 testing on unknown Domain 4 with multi-domain training, which adopts all known Domains (1,2,3) as the trained data.

Figure 10 shows the curves when using all known domains as the training set, and it can be observed from the training curves that the model has a limited effect in improving the accuracy of the unknown data of the testing domain (Domain 4). It even leads to a slight decrease in accuracy. In cases where data with large inter-domain distances are already included, adding data with smaller inter-domain distances degrades the accuracy. This suggests that an influx of too much data from different sources exacerbates the complexity of training and is not conducive to robust representation extraction. This observation aligns seamlessly with the expectations derived from the introduced multi-domain training method with NSFE-MMD.

Figure 7, Figure 8, Figure 9 and Figure 10 visually present the iterative performance curve testing on unknown egg data, showcasing the adaptability and generalization prowess of the multi-domain training method. The experiments offer strong evidence that the use of NSFE-MMD for matching domain pairs with greater disparities within the training dataset significantly enhances the model’s ability to generalize. This method proves more effective than the random selection of training data for mitigating domain shift, thereby highlighting the practical utility of the proposed approach.

### 3.4. Quantitative Experimental Analysis

As illustrated in Table 4, the performance metrics of YOLOv5 when trained using different strategies across various domains. The use of a checkmark (✓) in the “Domain1”, “Domain2”, and “Domain3” columns indicates the inclusion or exclusion of specific domains in the training process. “SDT” refers to single-domain training, where the model is trained on one domain only; “MDT” stands for multi-domain training involving multiple domains; and “ADT” refers to all-domain training. Specifically, the YOLOv5 model trained by the multi-domain training method based on NSFE-MMD has a detection mAP of 86.6% on the egg data of the unknown testing Domain 4, which is an increase of 86%, 63.7%, and 8% compared to models trained on a single domain, and an increase of 4.7% compared to models trained on all egg data of training domains. Compared with the single-domain and common multi-domain training methods, the proposed method shows the best detection performance (mAP) in unknown Domain 4. The proposed method also exhibits stronger generalization performance using less training data when facing “ADT” with more training data.

To further verify the method’s effectiveness, another unknown Domain 5 is tested and the results are shown in Table 5. The experimental results also support the view of the paper. To be specific, the YOLOV5 model trained by the multi-domain trained method based on NSFE-MMD has a detection mAP of 87.9% on the egg data of the unknown testing Domain 5, which is an increase of 87.9%, 3.3%, and 7.9% compared to models trained on a single domain, and an increase of 50.6% compared to models trained on all egg data of training domains.

The simplicity of the proposed method makes it possible to integrate it into other object detection models. The typical object detection model YOLOv8 was also utilized to verify the proposed method’s effectiveness.

As illustrated in Table 6, the YOLOv8 model trained by the proposed multi-domain training method has a detection mAP of 88.8% on the egg data of unknown testing Domain 4, which is an increase of 67%, 5.7%, and 4.4% compared to models trained on a single domain and an increase of 3.7% compared to models trained on all domains. The experimental results further elucidate the efficiency of the proposed method in improving performance on unknown egg data detection of practical production.

## 4. Conclusions

This study introduces a novel multi-domain training strategy leveraging NSFE-MMD, significantly enhancing the performance of egg crack detection models in practical applications. By integrating this approach into the YOLOv5 and YOLOv8 models, we have streamlined the application process, eliminating the need for complex network adjustments or parameter tuning. Experimental results demonstrate that the YOLOV5 and YOLOV8 models trained with our new method achieved detection mean Average Precision (mAP) of 86.6% and 88.8%, respectively, on an unknown testing domain, showing a notable improvement over models trained on single domains and randomly selected domains. Additionally, we constructed a multi-domain egg sample dataset, providing a valuable resource for future research. These achievements not only validate the effectiveness of our multi-domain training method but also offer a new solution for crack detection in large-scale egg production.

Future research endeavors will focus on the utilization of more robust strategies to enhance the model’s detection accuracy in the egg data of unknown testing domains and extend its applicability to detecting other defect categories within eggs.

## Figures and Tables

**Table 1:** Multiple-origin domain egg samples data.

Dataset	Origin	Cleaning Status	Acquisition System	The Number of Samples
Domain 1	Wuhan	washed	static	289
Domain 2	Wuhan	unwashed	dynamic	1902
Domain 3	Wuhan	washed	dynamic	1319
Domain 4	Qingdao	unwashed	dynamic	2577
Domain 5	Nanyang	unwashed	dynamic	473

**Table 2:** The NSFE-MMD distance between different domains.

Domain Pair	NSFE-MMD
Domain 1–Domain 2	0.94
Domain 2–Domain 3	0.55
Domain 1–Domain 3	1.0

**Table 3:** Parameter configuration.

Configuration	Parameter
Development environment	Anaconda3 + Jupyter
CPU	8*Xeon Gold 6330
GPU	RTX A6000
Operating system	Ubuntu 18.04
Accelerated environment	CUDA 11.3, cuDNN 8.3.0

**Table 4:** The test performance on unknown Domain 4 of YOLOV5 trained with different methods. Bold indicates the best performance.

Method	Domain 1	Domain 2	Domain 3	Precision	Recall	mAP.5
SDT	✓			0.012	0.006	0.006
SDT	✓			0.316	0.381	0.229
SDT		✓		0.781	0.768	0.786
MDT		✓	✓	0.846	0.802	0.856
MDT	✓	✓		0.856	0.780	0.847
Ours	✓		✓	0.838	**0.828**	**0.866**
ADT	✓	✓	✓	**0.866**	0.739	0.819

**Table 5:** The test performance on unknown Domain5 of YOLOV5 trained with different methods. Bold indicates the best performance.

Method	Domain 1	Domain 2	Domain 3	Precision	Recall	mAP.5
SDT	✓			0.000	0.000	0.000
SDT		✓		0.711	0.846	0.846
SDT			✓	0.738	0.709	0.800
MDT		✓	✓	0.801	0.797	0.854
MDT	✓	✓		0.833	0.854	0.871
Ours	✓		✓	**0.844**	0.885	**0.879**
ADT	✓	✓	✓	0.242	**1.00**	0.373

**Table 6:** The test performance on unknown Domain 4 of YOLOV8 trained with different methods. Bold indicates the best performance.

Method	Domain 1	Domain 2	Domain 3	Precision	Recall	mAP.5
SDT	✓			0.230	0.563	0.218
SDT		✓		0.775	0.791	0.831
SDT			✓	0.754	0.793	0.844
Ours	✓		✓	**0.852**	**0.806**	**0.888**
ADT	✓	✓	✓	0.832	0.759	0.851

## Data Availability

The data presented in this study are available on request from the corresponding author, the datasets presented in this article are not readily available because the data are part of an ongoing study.
